# Impact of snow manipulation on overwintering disease and frost damage across pasture grass species

**DOI:** 10.1038/s41598-025-21885-8

**Published:** 2025-10-30

**Authors:** Seiji Shimoda

**Affiliations:** NARO Hokkaido Agricultural Research Center Memuro Station (NARO/HARC/M), Memuro, 082-0081 Japan

**Keywords:** Snow manipulation, Soil temperature, Overwintering damage, Disease severity, Frost resistance, Pasture species, Plant ecology, Grassland ecology

## Abstract

Snow manipulation regulates soil temperature, reducing overwintering diseases and supporting pasture growth, though it has a limited effect on supporting grass growth. This study assessed the snow manipulation effects on overwintering damage and disease control in timothy (TY), orchardgrass (OG), and perennial ryegrass (PR). Snow manipulation decreased Mild-T days (soil temperatures near 0 °C), which correlated with reduces disease severity, particularly in TY. In plots with < 20 consecutive Mild-T days, maximum disease severity was lowest in TY (0.13) compared with that in OG (1.78) and PR (1.63). However, total overwintering damage also depended on disease and frost resistance. Although snow mold reduction did not consistently decrease damage, PR exhibited severe frost damage, making it highly vulnerable to snow manipulation. Contrastingly, OG compensated for overwintering losses with strong regrowth, maintaining similar dry matter yield. Snow manipulation effectively controls overwintering diseases but may raise frost damage in less cold-resistant species. TY showed the greatest adaptability, PR was highly frost-susceptible, and OG maintained productivity despite overwinter losses. These findings highlight the need to consider disease and frost resistance when using snow manipulation for disease control.

**Supplementary Information:**

The online version contains supplementary material available at 10.1038/s41598-025-21885-8.

## Introduction

Decadal climate change-induced snow cover reduction often increases the severity of frost damage (e.g., heaving and desiccation) owing to the continuation of snow-free periods and freeze-thaw cycles^1^. Additionally, delayed spring regrowth can occur because of the delayed thawing of frost soil after snowmelt and the delayed soil temperature increase caused by shallow winter snow cover and soil frost in overwintering crops^2–4^. Frost damage from heaving and desiccation typically occurs during late winter or early spring. Frost heaving occurs when saturated soils freeze with little or no snow cover. A reduction in seasonal snow accumulation may have a negative impact on the winter survival of overwintering vegetation, especially in regions where crops rely on adequate snow cover to survive harsh winters^5^. Various indices assess overwintering conditions, with cumulative negative temperatures serving as a typical frost damage index for overwintering crops^6,7^.

Temperate grass pastures introduced to Hokkaido, Northern Japan, often endure severely cold winters. Notably, orchardgrass (OG; *Dactylis glomerata* L.) and perennial ryegrass (PR; *Lolium perenne*) experienced extensive frost damage in the mid-1970s^8,9^, prompting a shift toward winter-hardy timothy (TY; *Phleum pratensis* L.), which increased from approximately 50% to 90% of the seeding ratio over 30 years. The latest seeding ratio of TY decreased again to 76%^10^, reflecting adaptation to summer warming.

Although a thick snowpack can protect overwintering plants from frost damage, it can also reduce the subsequent growth of plants owing to infection by pathogens (e.g., snow mold). Snow mold, a disease experienced by grasses and winter cereals exposed to snow in cold-temperate regions caused by several cold-adapted fungi, attacks overwinter grasses and crops in the Northern Hemisphere^11,12^. Persistent snow cover insulates the soil, increases the contact of leaf surfaces with the soil, and creates a dark, humid environment favorable for snow mold development^12^. The key characteristic of snow mold fungi is the ability to spread at temperatures near 0 °C under snow cover. The number of snow cover days is a well-known environmental indicator of snow mold^13^ because the temperature under the snow is maintained at 0 °C during thick snow cover^14^. Currently, snow mold control relies on chemical pesticides for disease control in winter wheat and turf. The chemical use of pasture grass is difficult from the viewpoints of cost and environmentally friendly management of forage use. Therefore, sustainable physical control methods based on an understanding of the environmental responses of snow mold are needed.

To investigate the effects of snow manipulation on pasture overwintering, two distinct methods—snow removal and snow compaction—were employed to monitor changing snow depths and temperature regimes^14–16^. Snow compaction alters the physical properties of the snowpack, notably increasing its density, which in turn influences thermal conduct and results in reduced soil temperatures^17,18^. Such reductions in soil temperature following snow compaction can delay the growth of winter wheat^2^ and regulate snow mold disease^14^. Snow removal, by eliminating the insulation, is anticipated to induce equal or more pronounced changes in soil temperature compared to compaction.

In this study, we hypothesized that controlling soil temperature through snow manipulation could decrease overwintering diseases and improve pasture growth. The first objective of this study was to determine the temperature required to control the severity of snow mold. Next, we considered whether snow mold control simultaneously causes frost heaving and freezing death owing to low temperatures. The optimal approach involves determining effective temperatures for pasture grass survival and selecting appropriate snow manipulation methods while acknowledging inevitable frost damage. In Hokkaido, farmers use agricultural machinery to regulate snow depth over large areas to freeze volunteer potatoes^15^. Snow manipulation is a feasible agricultural management option for local farmers^16,19^, and this study contributes to its practical application. Frost damage and snow mold, which tend to develop in low-temperature areas, are rarely studied separately, leaving their environmental dependencies poorly understood.

## Methods

### Site description and meteorological data collection

The study site was located at the Memuro Experiment Station of the Hokkaido Agricultural Research Center (NARO/HARC) (42.888 N, 143.074 E; elevation 94 m above sea level) in Memuro, Eastern Hokkaido, Japan. The annual mean air temperature and rainfall measurements taken at the station were 6.4 °C and 945 mm, respectively, from 2012 to 2021^20^. The region has a humid continental climate (Köppen Dfb, hemiboreal) and features well-drained volcanic ash soil (Andosol). The number of days with mean air temperatures below − 10 °C was 17 in 2018/19, 24 in 2019/20, and 22 in 2020/21.

To monitor the soil temperatures during winter, we installed thermometers with data loggers (Thermo buttons 22 L; Plug & Track Inc., Willems, France) at 2-h intervals from November 2018 in each block at depths of 0.02 m. In the no-treatment field area, a snow depth meter (Model SDM-311; Niigata Electric Co., Niigata, Japan) certified by the Japan Meteorological Agency was used to measure snow depth at 09:00 JST. For the snow compaction and snow removal treatments, we measured snow depth using a scale before and after treatments or every 10 d.

### Snow mold and freezing index

Environmental indicators for snow mold and frost damage include the number of snow cover days. Gray snow molds, such as *Typhula ishikariensis* S. Imai^21^, thrive in deep snow regions with extended snow cover^22^. Shimoda et al.^14^ suggested that snow mold is greater with longer periods at soil temperatures near 0 °C. *Sclerotinia borealis* Bubák & Vleugel prevails on plants that are predisposed to cold and are subsequently covered with thick snow. This study defines a ‘mild temperature’ (Mild-T) index as the number of days with a daily mean soil temperature of 0 ± 0.5 °C. Mild-T serves as a general indicator for various snow mold species, which require a transition period at 0 °C after exposure to lower temperatures. Cumulative freezing index (CFI) is widely used to assess cold stress, representing the cumulative degree-days below 0 °C from July 1 to June 30 of the following year. The CFI is also a reasonable environmental indicator of the occurrence of *Sclerotinia borealis*, which requires a period of cold exposure before snow accumulation.

### Disease and frost damage severity

To determine disease severity, we investigated pastures that overwintered in each plot 2 to 3 weeks after snowmelt. Causal pathogens were identified based on disease symptoms, and plant damage was evaluated specifically for fungal infections. Two snow mold pathogens, *T. ishikariensis* and *S. borealis*, were found in the field. Plant damage was scored individually using the following scale: 0 = no damage, 1 = half of the leaves dead, 2 = all leaves dead, 3 = less than half of the shoots dead, and 4 = all shoots dead.

### Snow compaction and removal practices

Our experimental design allowed for environmental control and variety validation in agronomic plots of approximately 10 m^2^ in the field. Experiments were conducted using a randomized complete block design with three replicates. A total of 4 × 3 plots were established: untreated control (Cont), snow compaction completed within 20 days after the first snowpack (SC1), intermittent compaction until 15 Feb (SC2), and snow removal (Rem). In the SC1 plots, the first snow compaction was performed within 5 d after the snow depth exceeded 0.05 m, followed by zero to one additional compaction operation within the subsequent month. In contrast, the SC2 plots involved four to five snow compaction operations conducted following the initial snowfall. Snow was compacted using a tractor (EDR-PKCPS6; Massey Ferguson, Beauvais, France) equipped with tire pressure rollers (600/65R38; Agri-index Co., Memuro, Japan). Snow removal, which is generally impractical for field crops owing to increased frost injury risk after snow cover loss^23^, was performed using a wheel loader (WA30; Komatsu, Tokyo, Japan). To minimize direct plant damage, snow compaction preceded removal in Rem plots. The snow was compacted and removed in the east–west direction, with a width of approximately 3.5 m. Based on preliminary trials^24^, we designed the snow compaction schedule as follows: after approximately 0.1 m depth of snow, snow was compacted once or twice in the SC1 plots and until the snow depth exceeded 0.50 m in the SC2 plots. Most treatment dates aligned with those reported for winter wheat fields from 2019 to 2021^14^. Most snow removal was performed after more than 0.1 m new snow cover. Natural and compacted snow were collected using an aluminum snow survey tube (inner diameter 50 mm, Climate Engineering Co., Niigata, Japan) and a linear soil sampler with a 30-mm internal diameter (04.04.00.30.C and 0.1.10.11.C; Eijkelkamp Co., Giesbeek, the Netherlands), and measured using an electronic scale (HL-300WP, A&D Co., Tokyo, Japan). Snow weights were taken inside an acrylic case to block the wind at the field. Snow density was determined by snow weight and depth. In plots where the snowpack depth was less than 0.05 m, accurate measurement of snow water equivalent was challenging owing to the fragility and discontinuity of the snow layer. Consequently, snow density could not be obtained for shallow snowfall and snow removal treatments.

### Pasture species and growth investigation

The test pasture species were TY, OG, and PR. Each species included early, medium, and late growth varieties: ‘Kunpuu’ (KUN), ‘Natsuchikara’ (NCH), and ‘Natsupirika’ (NPI) for TY; ‘Esajiman’ (ESA), ‘Harujiman’ (HAR), and ‘Toyomidori’ (TOY) for OG; and ‘Chinita’ (CHI), ‘Poroko’ (POR), and ‘Douto 1’ (DOU) for PR, respectively. These grass varieties are widely cultivated in Hokkaido, Japan. In perennial grass, summer climate affects root development and nutrient accumulation progress^25^, which changes the cold tolerance. To mitigate the effects of annual changes in cold tolerance. Seeds were sown at 20 g m^–2^ by hand in late August of each year.

The impact of snow mold on forage grass crops is difficult to assess because of the regenerative ability of grasses following biotic and abiotic stresses^26^. Therefore, we used first-cut aboveground production as a growth index after overwintering. A 1.0 × 2.0 m section was harvested, fresh weight was measured using a scale (SJ-WP; A&D Co., Tokyo, Japan), and a 200 g sample was dried for 1 week at 80 °C to estimate dry matter production. Rapid post-snowmelt growth is essential for early grazing, influencing first-cut hay yield at the heading stage. The heading date was recorded as the first occurrence of heading within a 1.0 × 2.0 m area at the center of each plot (*n* = 3).

### Statistical analyses

All analyses were conducted using R v. 4.3.2 software. Differences in disease and frost damage severity and dry matter production among treatments were tested. First, a one-way analysis of variance (ANOVA) was applied to determine whether there were significant differences among treatments. After ANOVA resulted in significant results (*p* < 0.05), statistical differences were evaluated using Tukey’s honestly significant difference test (*p* < 0.05).

## Results

### Environmental conditions during winter

**Fig. 1:**
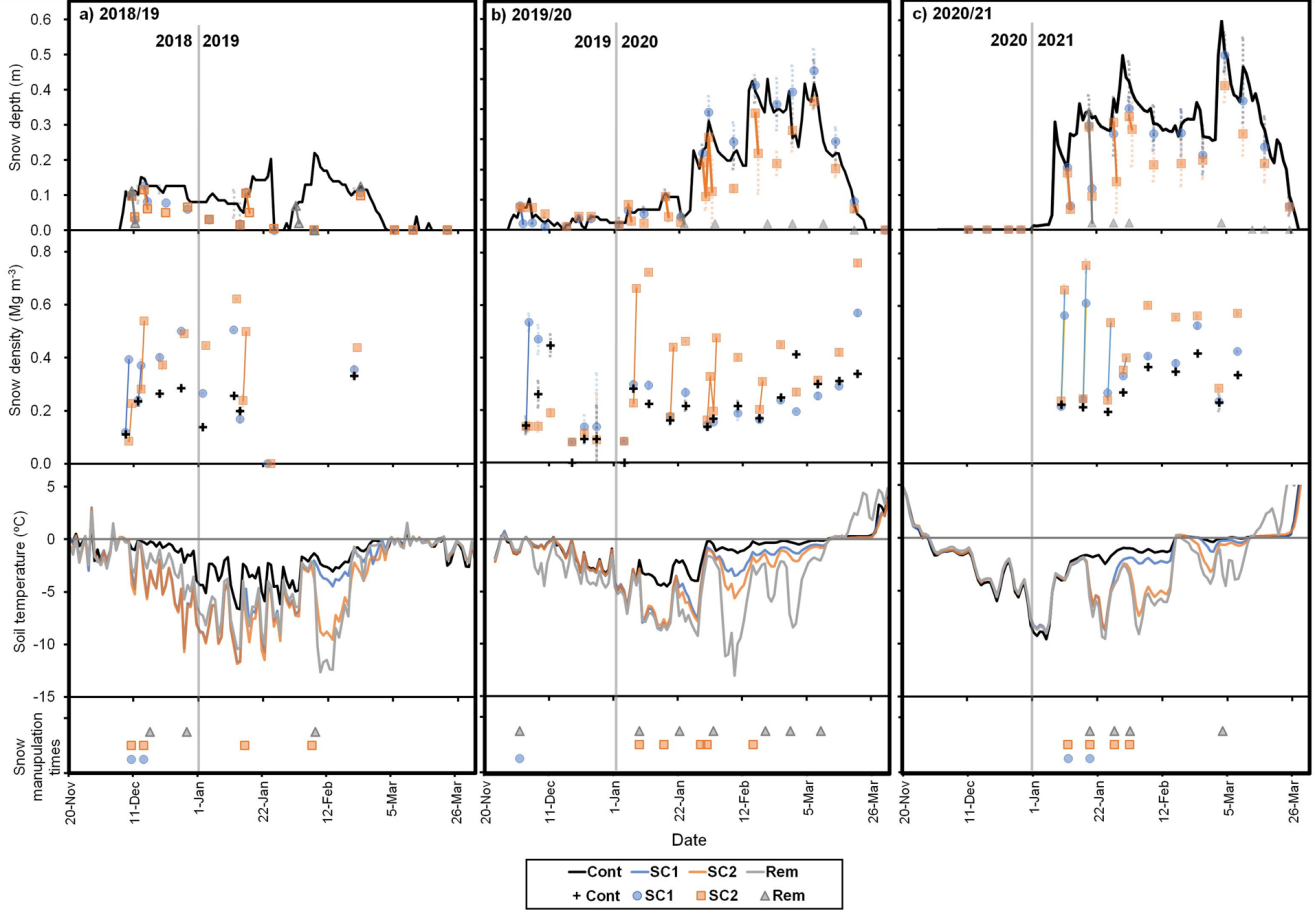
Temporal changes in snow depth, snow density, and soil temperature at depths of 0.02 m, with treatment dates for (**a**) 2018/19, (**b**) 2019/20, and (**c**) 2020/21. Solid lines represent changes in depth and density of snow before and after snow compaction. Error bars (dot lines) represent the standard error of the mean for snow depth and density (*n* = 3).

The years of low snowfall in December and January resulted in markedly lower soil temperatures, and snow compaction accelerated temperature reduction. The maximum snow depth was lower in the winter of 2018/19 than in other years (Fig. 1a). By midwinter, snow depth was nearly zero and remained below 0.20 m throughout winter, causing sharp soil temperature fluctuations regardless of treatment. In the winter of 2019/20, soil temperature rapidly decreased below − 10 °C in Rem. The difference in soil temperatures between the snow removal and compaction treatments was higher in 2019/20 (Fig. 1b) than in 2020/21 (Fig. 1c). Snow cover began later in the winter of 2020/21 than in the other years. The first snowfall of the year substantial altered the snow depth, reducing snow depth from 0.10 to 0.03 m in 2018/19, from 0.08 to 0.02 m in 2019/20, and from 0.18 to 0.07 m in 2020/21. Snow density increased by 3.0, 4.0, and 2.7 times, respectively. Compaction immediately after snow cover lowered soil temperature, and in the SC1 plots, soil temperature remained lower than in the Cont plots throughout the snow cover period. Snow insulation effects are achieved when natural snowfall reaches a depth of 0.2 to 0.3 m or more, but in 2018/19, due to shallower snow depth throughout the winter, soil temperature often dropped below − 5 °C even in the Cont plots. Under conditions of substantial snowpack, the effectiveness of snow compaction decreases, resulting in a final compaction ratio that is significantly lower than the initial value. In the SC2 plots, snow density increased by only 1.1 to 2.0 times following the final compaction.

### Damage severity

**Fig. 2:**
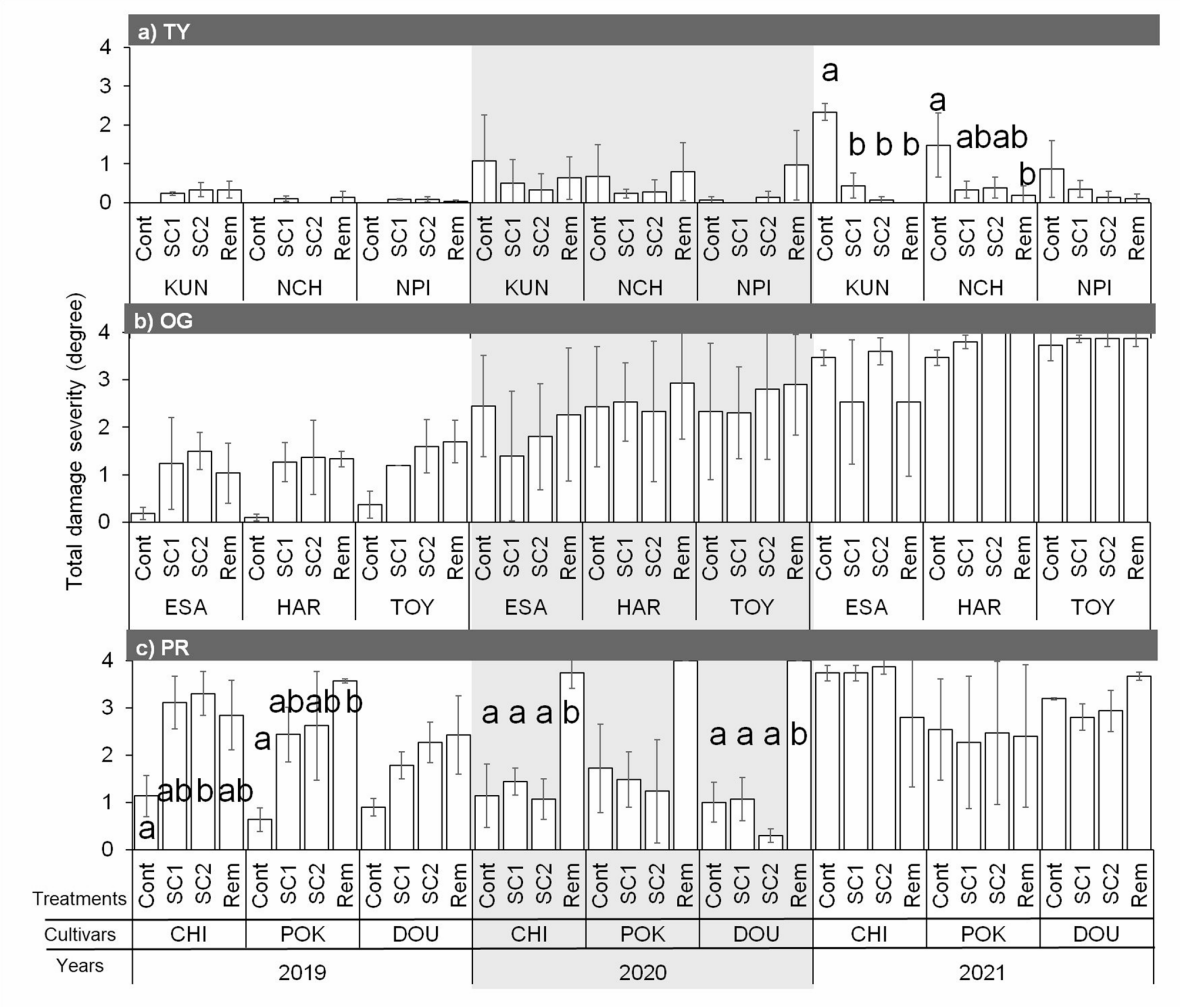
Total damage severity in (**a**) TY, (**b**) OG, and (**c**) PR for 3 years under different snow manipulation. Cont, control; SC1, snow compaction completed within 20 days after the first snowpack; SC2, intermittent snow compaction until 15 Feb; Rem, snow removal throughout winter. Error bars represent the standard error of the mean (*n* = 3). Different letters denote significant differences among treatments and between years (*p* < 0.05, Tukey’s honestly significant difference test).

Overwintering damage in TY was minimal in 2019, despite the short snow cover period. Conversely, in 2021, the KUN and NCH varieties showed significantly higher damage severity in the Cont plots than in the other treatments (Fig. 2a). At the OG site, no significant difference was observed among the treatments (Fig. 2b). In PR, compacted and snow-removed plots showed significantly higher damage in POK in 2019, DOU in 2020, and CHI in both 2019 and 2020 (Fig. 2c).

**Fig. 3:**
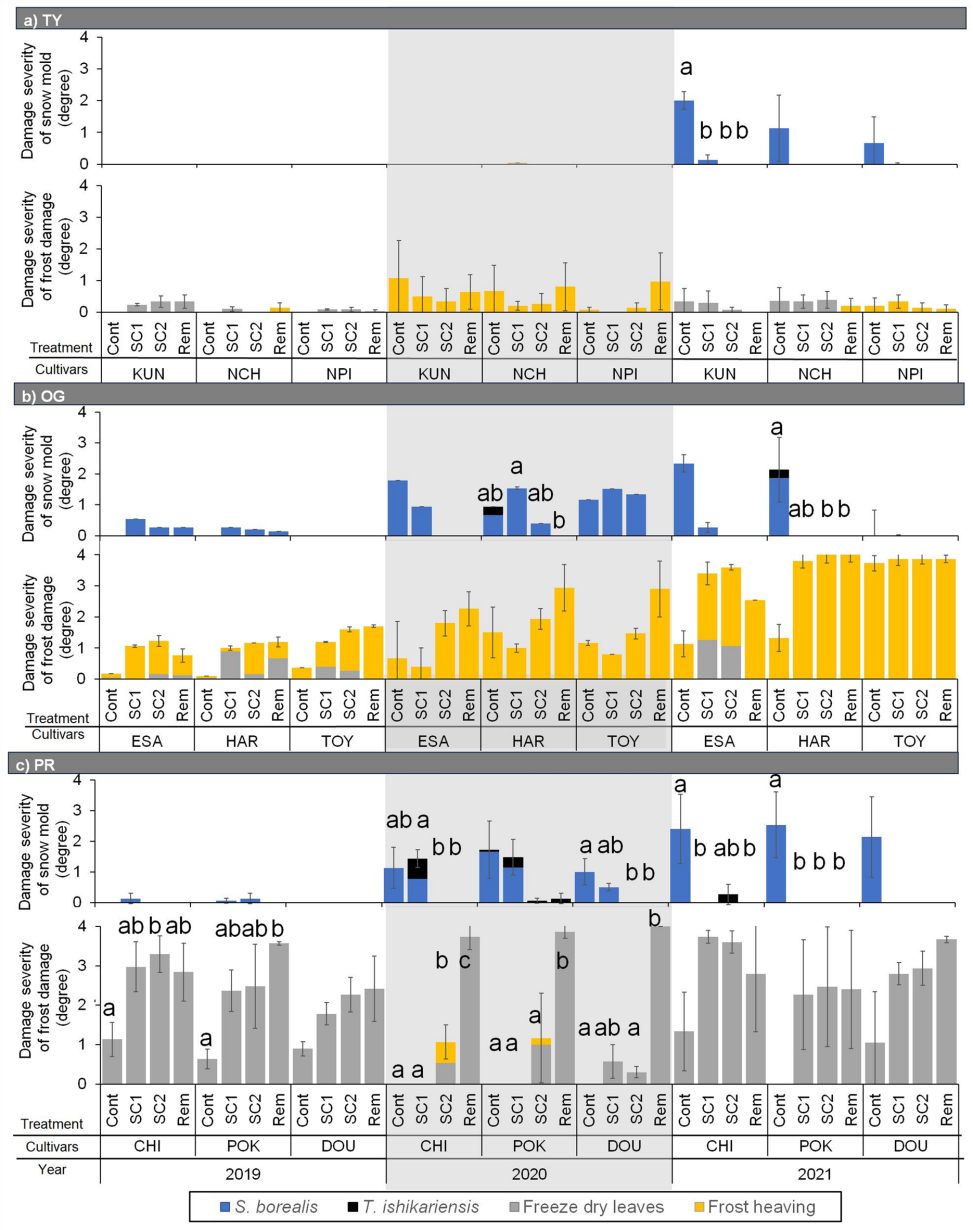
Disease severity and frost damage severity over 3 years in (**a**) TY, (**b**) OG, and (**c**) PR under different snow manipulation treatments: control (Cont), snow compaction completed within 20 days after the first snowpack (SC1), intermittent snow compaction until 15 Feb (SC2), and snow removal (Rem). Error bars represent the standard error of the mean for disease and frost damage severity (*n* = 3). Different letters denote significant differences among treatments and between years (*p* < 0.05, Tukey’s honestly significant difference test). Bar graphs are color-coded to differentiate between pathogens (*S. borealis* and *T. ishikariensis*) and frost damage types (freeze dry leaves and frost heaving).

Disease severity was low in 2019 across all treatments, remaining below 0.5 regardless of variety. In contrast, in 2021, the Cont plot had high disease incidence across all pasture species, with one TY, two OGs, and all PRs showing severity greater than 2 (Fig. 3). Disease severity was significantly higher in the Cont plot than in the snow-compacted plots, with removal treatments also reducing severity in one PR variety in 2020 and in one TY, one OG, and two PR varieties in 2021. *Sclerotinia borealis* was prevalent in most snow mold-infected plots. The severity of frost damage varied mainly by species, with frost heaving occurring more frequently in OG and freeze-dried leaves in PR. In OG, there was no significant difference in the extent of frost damage between treatment plots. However, in PR, frost damage occurrence differed significantly between the Cont and Rem plots for all varieties in 2020, in contrast to the single variety (‘POR’) affected in 2019.

**Fig. 4:**
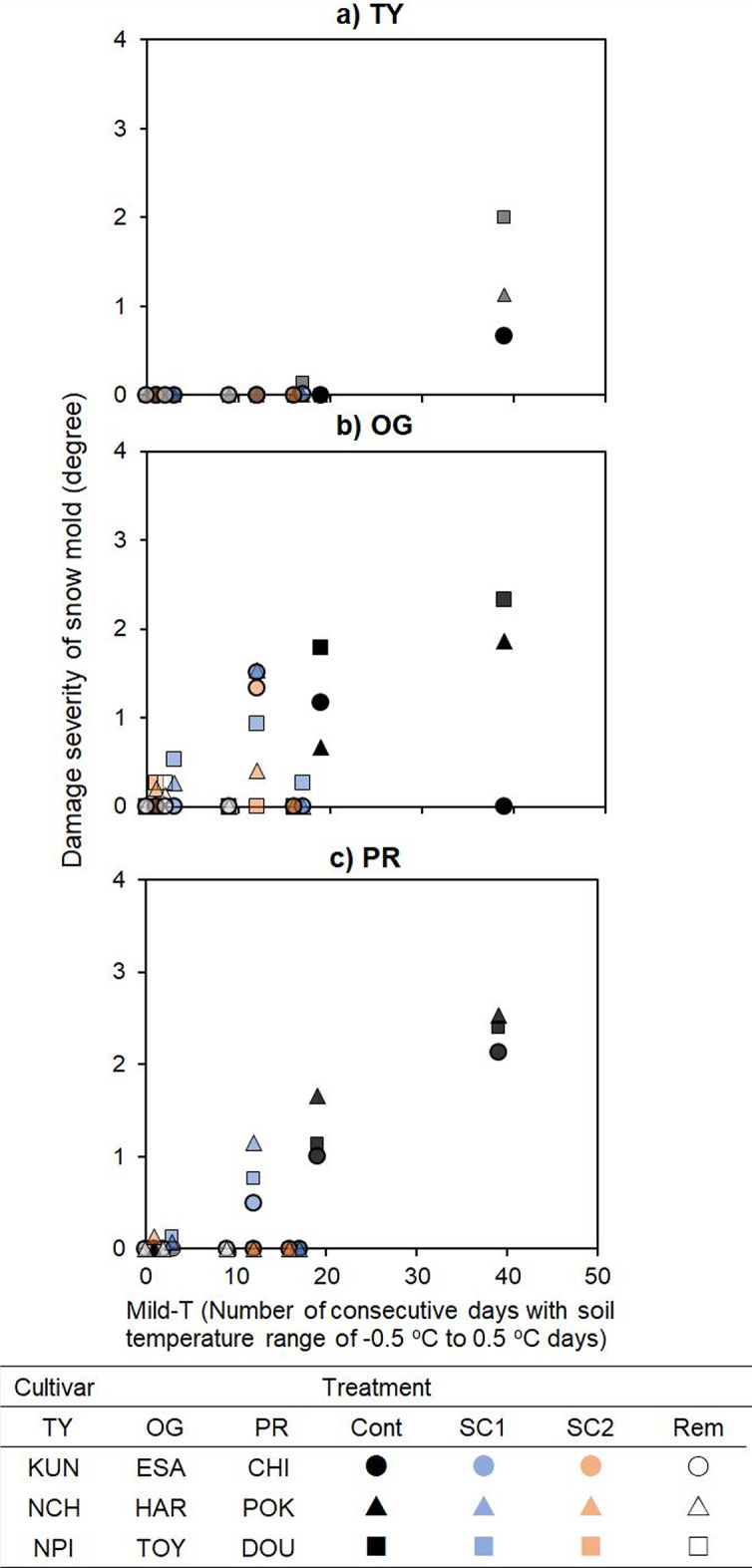
Relationship between disease severity in winter wheat cultivars and the number of days with a soil temperature range of − 0.5 °C to 0.5 °C. Plot colors represent different treatments (black: control (Cont); blue: snow compaction completed within 20 days after the first snowpack (SC1); orange: intermittent snow compaction until 15 Feb (SC2); white: snow removal (Rem)) over 3 years for (**a**) TY, (**b**) OG, and (**c**) PR. Varietal differences are shown in the form of plots. Square, triangle, and circle plots indicate ‘Kunpuu,’ ‘Natsuchikara,’ and ‘Natupirika’ in TY; ‘Esajiman,’ ‘Harujiman,’ and ‘Toyomidori’ in OG; and ‘Tinita,’ ‘Poroko,’ and ‘Doto 1’ in PR.

The plots experienced less than 20 consecutive days of Mild-T, except for the Cont plot in 2020/21 (Fig. 4). The response of disease severity to consecutive days of Mild-T showed a difference in disease severity among pasture species, and disease severity in TY was lower than that in OG and PR, especially for shorter days of Mild-T. When Mild-T lasted fewer than 20 consecutive days, disease severity remained below 0.13 in TY, while it reached 1.78 in OG and 1.63 in PR. Severity generally increased with prolonged Mild-T across most plots, though the ESA of OG showed a decreasing trend. We also observed that the CFI and Mild-T index showed similar trends, with cooler soil temperatures resulting in lighter snow mold damage. Snow mold rarely occurred when CFI was below 200 °C in TY, while OG had slightly higher severity than PR under similar conditions (Appendix A1a). Additionally, significant frost damage in OG and PR was observed, although the relationship with the CFI was unclear (Appendix A1b).

### Growth speed and production

**Fig. 5:**
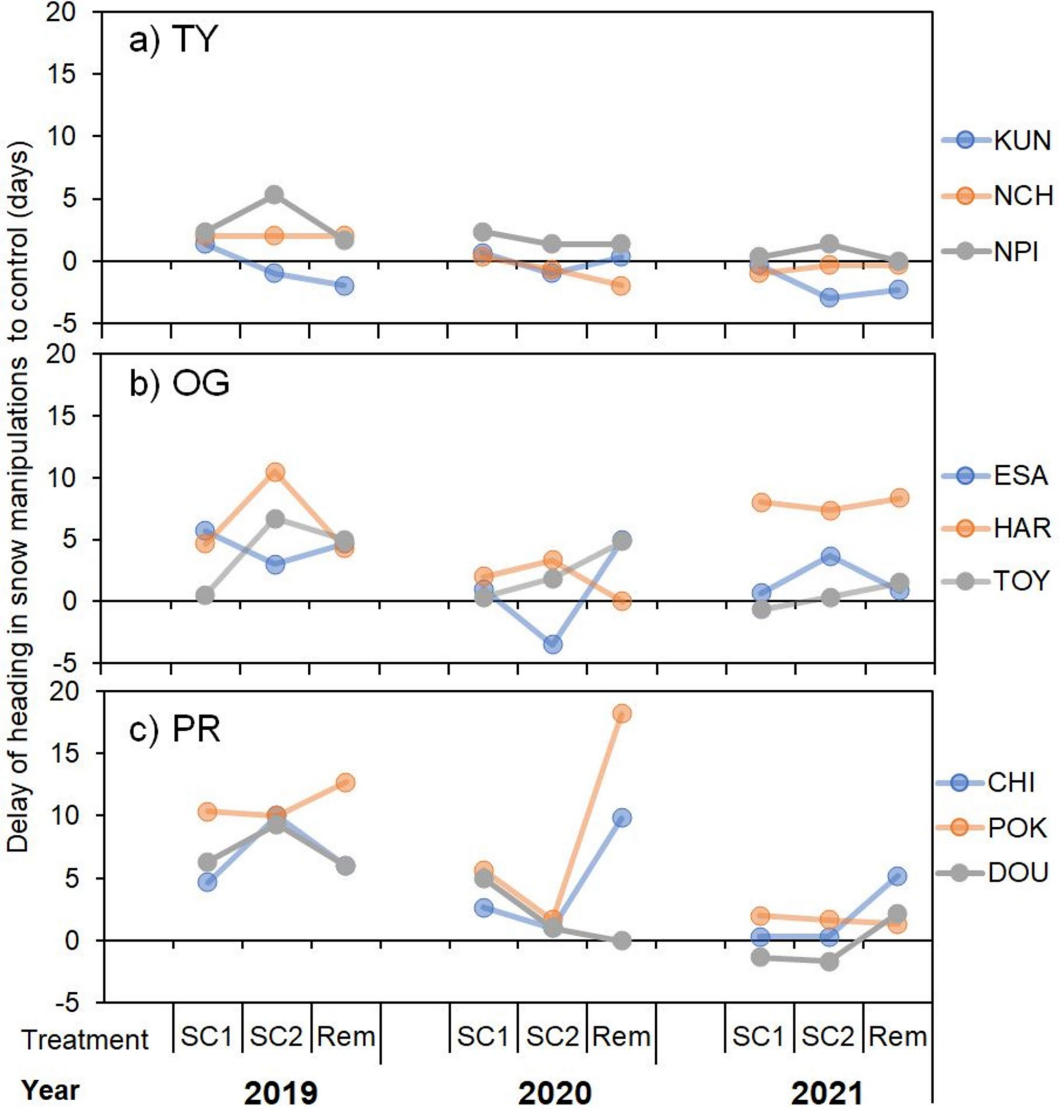
Delay of heading in snow manipulations to control in (**a**) TY, (**b**) OG, and (**c**) PR.

Growth speeds at the TY site were less affected by snow manipulation than those at the OG and PR sites. Additionally, growth delay was related to low temperatures due to snow manipulation in PR. The heading period in the Cont plots was similar to that in the snow manipulation plots, and 11 of the 27 (40%) snow manipulation plots had earlier headings than the Cont plots (Fig. 5a). In OG and PR, most varieties showed later heading in the manipulation plots than in the Cont plots (Fig. 5b, c), with delays exceeding 10 d in POK of OG and HAR of PR.

**Fig. 6:**
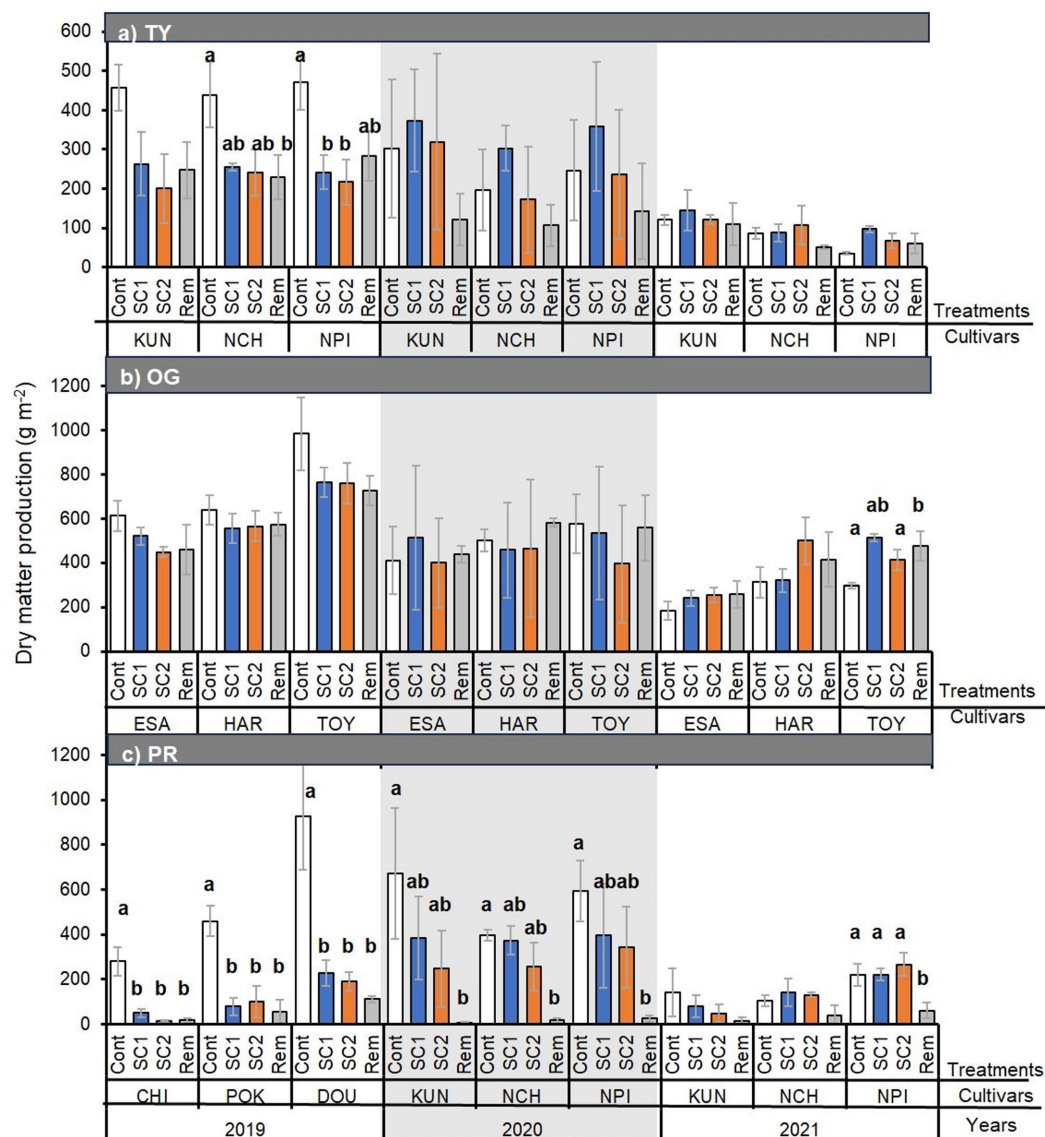
Dry matter production under different snow manipulation treatments: control (Cont; white bars), snow compaction completed within 20 days after the first snowpack (SC1; orange bars), intermittent snow compaction until 15 Feb (SC2; gray bars), and snow removal (Rem). Error bars represent the standard error of the mean (*n* = 3). Different letters denote significant differences among treatments and between years (*p* < 0.05, Tukey’s honestly significant difference test).

In TY, no difference in production was observed among the treatments in 2020 and 2021 (Fig. 6a). Dry matter production in OG was similar among treatments, except for ‘TOY’ in 2021 (Fig. 6b). The adverse effect of snow manipulation on dry matter productivity was largest in PR, and most varieties showed significantly higher production in the Cont plots than in the snow manipulation plots (Fig. 6c). In particular, snow removal had a substantial adverse effect on PR. The Rem plots exhibited less than 100 g m^− 2^ production across all years.

## Discussion

Snow manipulation effectively suppressed snow mold by regulating soil temperatures; however, significant frost damage unrelated to disease offset its positive effects on growth.

### Soil temperature regulation

Reduction in soil temperature is influenced by both ambient air temperature and snowpack characteristics. In years with shallow snow, soil temperature in the snow removal plots were similar to those in frequently compacted plots. Generally, when the snow depth exceeds a threshold of approximately 0.2 to 0.3 m, the thermal insulation of the snowpack effectively mitigates the influence of cold ambient air, thereby preventing significant declines in soil temperature^17^. The effect of snow management methods on soil temperature varies depending on the depth of snow cover. Snow removal directly alters thermal conductivity, often causing an abrupt drop in soil temperature^18^. In contrast, under deep snow conditions, compaction does not sufficiently increase snow density throughout the entire snow layer, resulting in only a moderate decrease in soil temperature. Currently, the density of snow after mechanical compaction remains insufficiently understood due to limited experimental data, which is available only from specific regions^14–19^. For a comprehensive understanding, future studies should include quantitative analyses that consider snow quality parameters such as moisture content in other regions.

### Reduction of snow mold

The effects of snow manipulation varied somewhat among the pasture species. The highly cold-tolerant species TY avoided frost injury, whereas low temperatures from snow manipulation nearly eliminated snow mold. The response of disease severity to consecutive days of Mild-T indicated that TY had a higher overwintering tolerance than OG and PR. The effectiveness of snow manipulation in reducing snow mold in OG and PR was consistent with previous findings in wheat^19^, suggesting that maintaining soil temperatures at 0 ± 0.5 °C for over 30 d is a threshold for fatal damage (severity > 2). *Sclerotinia borealis* requires temperatures of − 2 to − 3 °C followed by heavy snow to develop^15^. Snow manipulation shortened periods near 0 °C, limiting the conditions favorable for snow mold pathogens. Across species, snow manipulation showed a declining trend in disease severity.

Frost tolerance of the varieties is related to the decrease in moisture content and increase in nutrient concentration in the hardened tissue, which are cold-induced physiological changes^27^. A previous study revealed the order in resistance to *S. borealis* was in accordance with their specific freezing resistance; PR, with lower cold tolerance, is more prone to fungal damage than more freeze-resistant species such as TY^13^. Our results showed disease severity correlates more directly to Mild-T than the CFI, indicating that a cold environment is not a prerequisite for disease development.

Differences in disease response to Mild-T suggest overwintering disease tolerance in each species; however, few varietal differences were detected. Some varieties may differ in their thermal response to disease; however, reductions in disease often coincided with increased frost damage, making total damage severity dependent on both disease resistance and frost resistance. Varietal differences in frost resistance is often influenced by pre-winter growth management^7^, contributing to uncertainty in the relationship between Mild-T and disease severity. Changes in snow mold occurrence with snow compaction indicated the effectiveness of Mild-T as a soil temperature indicator for disease risk assessment.

### Frost damage by snow manipulation

The difference in the degree of frost damage depended mainly on the species. Low-temperature damage from snow manipulation was the primary cause of the reduced growth at the PR and OG sites. Even in 2019, a year of low snow mold incidence, snow manipulation significantly reduced productivity at these sites. The adverse effects of low temperatures were more pronounced in PR than in OG. Since OG generally has greater cold tolerance than PR^9^, our results reflect the general overwintering characteristics of pasture species.

In our experiment, frost heaving was the major form of frost damage in OG. Frost heaving can damage plant roots in pastures, especially under conditions with inconsistent freezing and thawing cycles around 0 °C. In Hokkaido, breeding efforts have focused on developing OG varieties with strong spring regrowth to compensate for overwintering losses^27^. In this study, the spring regrowth of OG mitigated the effects of cold injury due to snow manipulation.

Our results lead us to consider the following question: Can snow cover be artificially managed for pasture management? A previous study indicated that snow compaction can control snow mold without causing yield loss in cold-tolerant wheat varieties^14^. Snow removal is used in turf management to suppress gray snow mold, which requires prolonged snow cover^11^. However, in pasture grass, the priority should be in evaluating its agronomic feasibility and potential benefits for forage production rather than just disease suppression.

Pasture grass is a perennial crop; consequently, growth delays owing to snow manipulation can result in shorter growing periods and lower annual yields. In cold-tolerant species, such as TY, snow compaction is a relatively effective method for preventing fatal overwintering diseases without apparent growth delays. In OG, growth delays did not contribute to the loss of first-cut dry mass production. Previous studies on winter wheat showed that low spring soil temperatures in snow-compacted fields delayed heading by several days; however, subsequent growth compensated for the loss of spring dry matter production^3^. Similarly, at the OG sites, high regrowth ability compensated for overwinter loss, resulting in no significant difference in dry matter production. For pastures containing cold-sensitive species, snow manipulation offers no clear agricultural benefit. Improved cold hardiness and cultivation methods may increase the chances of successful snow manipulation. Improved cold-sensitive varieties or cultivation may increase the likelihood of successful snow manipulation. In general, survival depends on pre-wintering growth; therefore, the potential of snow manipulation to improve pre-wintering growth needs further investigation.

### Snow manipulation as a pasture management strategy

Cold-sensitive pasture species lack sufficient resistance to temperatures below − 10 °C, making overwintering cold damage a key concern before considering snow mold mitigation. In such cases, avoiding intervention may be the best strategy to maximize production. At the study site, daily mean air temperatures fell below − 10 °C for 17 to 24 d. Historically, this region has been at the survival threshold for both OG and PR^8,9^, and the addition of colder winters to current climatic conditions further threatens healthy growth. In such a cold region, snow removal can reduce snow mold damage but also increases the risk of fatal frost damage, particularly in PR owing to its high frost sensitivity. This study discussed pasture adaptation to snow manipulation under current climatic conditions. Global warming has led to rising temperatures in spring and early winter, leading to extreme snowpack patterns in snow-covered regions worldwide^28^, including Northern Japan^29^. Some studies have indicated that snowfall decreases the risk of winter injury through potential soil heaving and ice encasement^7^. Identifying optimal temperature ranges for winter management can inform future agricultural adaptations to climate change.

## Conclusions

The study attempted to effectively physically control snow manipulation for the overwintering of pasture grasses without fatal disease infection and frost damage. Snow was manipulated through compaction and removal to control the snow depth and thermal insulation, and soil temperature decreased after practice. Our findings demonstrated that manipulating snow cover significantly alters soil temperature dynamics, reducing the number of days with near-freezing temperatures, an important factor in overwintering disease progression. This reduction in thermally conducive conditions for pathogens led to lower disease severity, particularly in TY, which exhibited the highest adaptability to snow manipulation. However, our results also revealed that the benefits of disease reduction must be weighed against potential increases in frost damage, particularly in frost-sensitive species such as perennial ryegrass. The thermal index of Mild-T showed clear differences in the severity of disease among species, while cold damage was unstable in its response to temperature. By integrating soil temperature modulation with plant resilience traits, our study advances the mechanistic understanding of plant responses to winter stress and contributes to broader discussions on optimizing plant production systems under variable climatic conditions.

## Supplementary Information

Below is the link to the electronic supplementary material.

Supplementary Material 1

Supplementary Material 2

## Data Availability

Data is provided within the manuscript or supplementary information files.
