# Supplementary figures and images for: Impact of snow manipulation on overwintering disease and frost damage across pasture grass species
Source: Sci Rep. 2025 Oct 30;15:38038. doi: 10.1038/s41598-025-21885-8 (PMC12575813; doi:10.1038/s41598-025-21885-8)

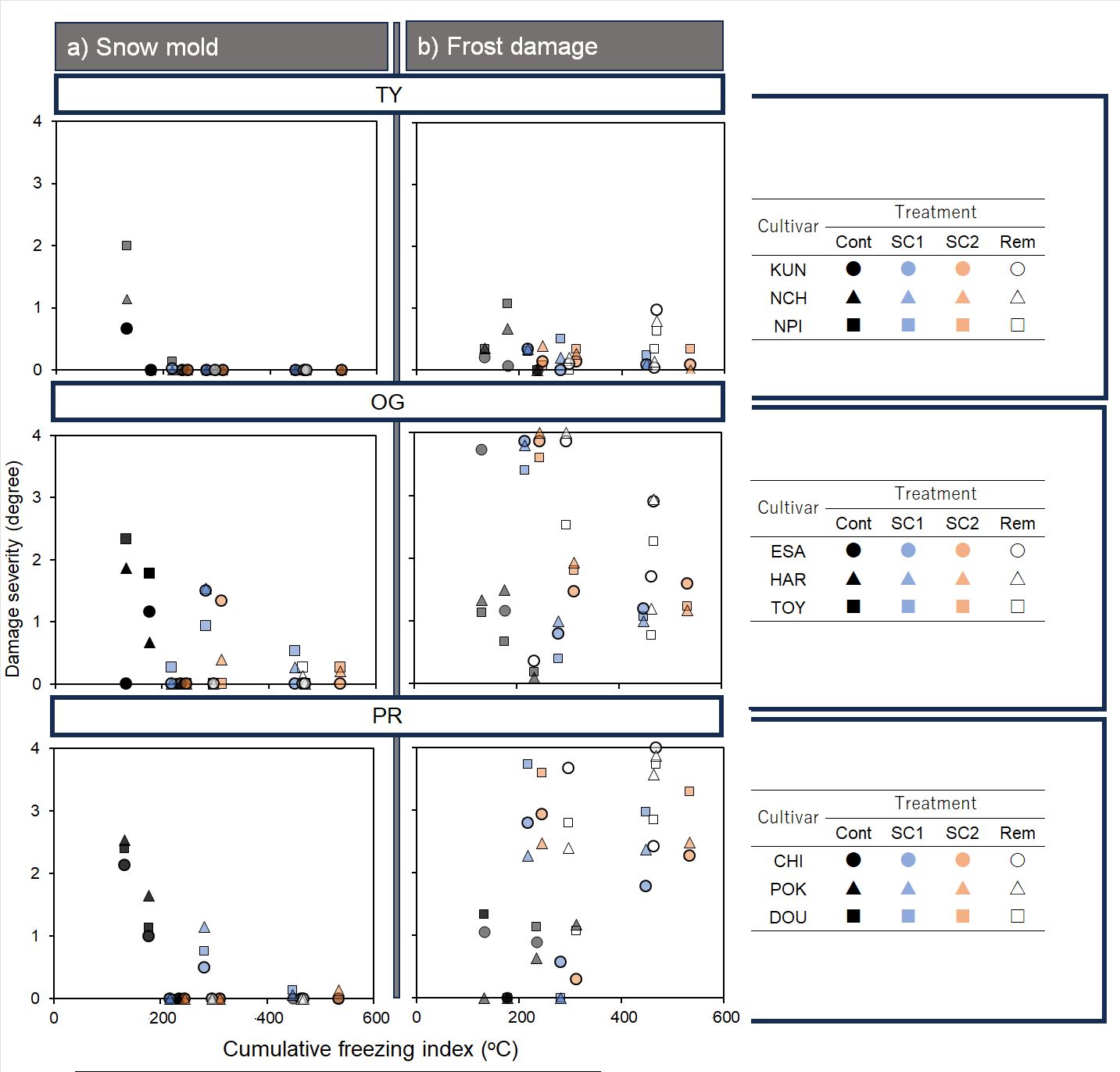

Supplement: Supplementary file 1 — Supplementary Material 1 [file 41598_2025_21885_MOESM1_ESM.jpg]
