# Supplementary material for: Impact of snow manipulation on overwintering disease and frost damage across pasture grass species
Source: Sci Rep. 2025 Oct 30;15:38038. doi: 10.1038/s41598-025-21885-8 (PMC12575813; doi:10.1038/s41598-025-21885-8)
Supplement: Supplementary file 2 — Supplementary Material 2 [file 41598_2025_21885_MOESM2_ESM.docx]

**Appendix A1** Relationship between the CFI and frost damage severity of a) snow mold and b) frost damage in winter wheat cultivars. Plot colors indicate differences in treatments (black: control (Cont); blue: snow compaction completed within 20 days after the first snowpack (SC1); orange: intermittent snow compaction until 15 Feb (SC2); white: snow removal (Rem)) over 3 years for TY, OG, and PR. Varietal differences are shown in the form of plots. Square, triangle, and circle plots indicate ‘Kunpuu,’ ‘Natsuchikara,’ and ‘Natupirika’ in TY; ‘Esajiman,’ ‘Harujiman,’ and ‘Toyomidori’ in OG; and ‘Tinita,’ ‘Poroko,’ and ‘Doto 1’ in PR.
